# Revealing the hidden biodiversity of Antarctic and the Magellanic Sub-Antarctic Ecoregion: A comprehensive study of aquatic invertebrates from the BASE Project

**DOI:** 10.3897/BDJ.11.e108566

**Published:** 2023-08-17

**Authors:** Sebastian Rosenfeld, Claudia S. Maturana, Melisa Gañan, Javier Rendoll Cárcamo, Angie Díaz, Tamara Contador, Cristian Aldea, Claudio Gonzalez-Wevar, Julieta Orlando, Elie Poulin

**Affiliations:** 1 Millennium Institute Biodiversity of Antarctic and Subantarctic Ecosystems (BASE), Santiago, Chile Millennium Institute Biodiversity of Antarctic and Subantarctic Ecosystems (BASE) Santiago Chile; 2 Cape Horn International Center (CHIC), Puerto Williams, Chile Cape Horn International Center (CHIC) Puerto Williams Chile; 3 Centro de Investigación Gaia‑Antártica, Universidad de Magallanes, Punta Arenas, Chile Centro de Investigación Gaia‑Antártica, Universidad de Magallanes Punta Arenas Chile; 4 Laboratorio de Estudios Dulceacuícolas Wankara, Programa de Conservación Biocultural Subantártica, Universidad de Magallanes, Puerto Williams, Chile Laboratorio de Estudios Dulceacuícolas Wankara, Programa de Conservación Biocultural Subantártica, Universidad de Magallanes Puerto Williams Chile; 5 Millennium Nucleus of Austral Invasive Salmonids - INVASAL, Concepción, Chile Millennium Nucleus of Austral Invasive Salmonids - INVASAL Concepción Chile; 6 FEHM-Lab (Freshwater Ecology, Hydrology and Management), Departament de Biologia Evolutiva, Ecologia i Ciències Ambientals, Facultat de Biologia, Universitat de Barcelona (UB), Diagonal 643, 08028, Barcelona, Spain FEHM-Lab (Freshwater Ecology, Hydrology and Management), Departament de Biologia Evolutiva, Ecologia i Ciències Ambientals, Facultat de Biologia, Universitat de Barcelona (UB), Diagonal 643, 08028 Barcelona Spain; 7 Institut de Recerca de la Biodiversitat (IRBio), Universitat de Barcelona (UB), Diagonal 643, 08028, Barcelona, Spain Institut de Recerca de la Biodiversitat (IRBio), Universitat de Barcelona (UB), Diagonal 643, 08028 Barcelona Spain; 8 Departamento de Zoología, Universidad de Concepción, Concepción, Chile Departamento de Zoología, Universidad de Concepción Concepción Chile; 9 Facultad de Ciencias, Centro FONDAP IDEAL, Instituto de Ciencias Marinas y Limnológicas (ICML), Universidad Austral de Chile, Valdivia, Chile Facultad de Ciencias, Centro FONDAP IDEAL, Instituto de Ciencias Marinas y Limnológicas (ICML), Universidad Austral de Chile Valdivia Chile; 10 Departamento de Ciencias Ecológicas, Facultad de Ciencias, Universidad de Chile, Santiago, Chile Departamento de Ciencias Ecológicas, Facultad de Ciencias, Universidad de Chile Santiago Chile

**Keywords:** Cape Horn Biosphere Reserve, freshwater ecosystems, GBIF, macroinvertebrates, Southern Ocean

## Abstract

**Background:**

Antarctica, its outlying archipelagoes and the Magellanic Subantarctic (MSA) ecoregion are amongst the last true wilderness areas remaining on the planet. Therefore, the publication, citation and peer review of their biodiversity data are essential. The new Millennium Institute Biodiversity of Antarctic and Subantarctic Ecosystems (BASE), a Chilean scientific initiative funded by the National Agency of Research and Innovation, contributes 770 new records of aquatic invertebrates as a point of reference for present-day biodiversity research at these latitudes.

**New information:**

The occurrence dataset presented here has never been released before and is the result of the systematic recording of occurrences of several taxa across the Antarctic, Subantarctic and Magellanic Subantarctic ecoregions. We collected data from marine and freshwater invertebrates across numerous samplings from 2008 to 2023. From the 770 occurrences, we identified 160 taxa, 125 at species level and 35 at the genus level. The database has been registered in the Global Biodiversity Information Facility (GBIF). The publication of this data paper was funded by the Belgian Science Policy Office (BELSPO, contract n°FR/36/AN1/AntaBIS) in the Framework of EU-Lifewatch as a contribution to the SCAR Antarctic biodiversity portal (biodiversity.aq)

## Introduction

The study and characterisation of biodiversity in the different ecosystems of the planet is a challenge and a task of grand proportions since it is estimated that the Earth is inhabited by about 10 million species, which requires a significant investment of funds and work to generate, collect and analyse biodiversity data (Troudet et al. 2017). In addition, due to the environmental crisis that ecosystems have experienced in recent decades and its diverse effects on biota, a global need for biodiversity data has been generated (Barnosky et al. 2011, Pecl et al. 2017). For this reason, data exchange practices and tools have been developed, such as the Global Biodiversity Information Facility (GBIF) and the Ocean Biodiversity Information System (OBIS), which follow the FAIR foundational principles (Wilkinson et al. 2016) that facilitate access and research process for species occurrence records. However, despite these international efforts devoted to the generation of data on biodiversity, even today, only a small proportion of global records are estimated to have been made available online (Ariño 2010, Maturana et al. 2019, Rosenfeld et al. 2022), with some critical knowledge biases detected in some taxonomic groups (Troudet et al. 2017, Rocha‐Ortega et al. 2021, Llorente-Culebras et al. 2023).

The Southern Ocean (SO) surrounds Antarctica and is one of the main drivers of global ocean circulation (Rintoul and Garabato 2013). Regarding biodiversity, the distribution of organisms in the SO seabed ecosystems largely reflects the complex interplay of geological, oceanographic and biological elements through space and time (Griffiths et al. 2009) Interpretation of these distributions can help to understand better the role of the SO in the ecological processes of the Southern Hemisphere and project into the future the changes in the distributions of biota due to the effect of global change (Griffiths et al. 2009, Lopez-Farran et al. 2021). From another perspective, Antarctica and the SO have been under the Antarctic Treaty System since 1961, in which signatory nations agree to prioritise current and future management and protection of the continent's biodiversity and ecosystem values. Comprehensive protection of the Antarctica environment cannot be addressed without a complete, unbiased and systematic publication of biodiversity data ensuring the accomplishment of such a crucial endeavour (Wauchope et al. 2019, Hawes et al. 2023).

It is essential to highlight that, despite the research efforts in Antarctica and the SO in the last decades in generating knowledge in biodiversity, there are still many poorly-sampled and -studied areas and habitats. In this regard, the intertidal and shallow subtidal environments of Antarctica and Subantarctic environments are considerably less well-sampled than either the surrounding deep sea (Brandt et al. 2014, Ojeda et al. 2014, Griffiths and Waller 2016, Rosenfeld et al. 2017, Aldea et al. 2020, Jossart et al. 2023). Likewise, in continental environments, recent studies have identified important sampling gaps in Antarctica and Subantarctic ecoregions (Chown and Convey 2016, Maturana et al. 2019, Ganan et al. 2021, Hawes et al. 2023). The Subantarctic environments represent a large and remote geographic area and, due to its extreme conditions (climatic and oceanographic) (Griffiths and Waller 2016), it denotes a challenge when undertaking biodiversity inventories. For example, in the Magellanic Subantarctic ecoregion, it has been described that the diversity of representative and moderately known groups, such as molluscs, would be underestimated (Aldea et al. 2020, Rosenfeld et al. 2020). In addition, with the progress of molecular tools to date, new lineages continue to be detected in both invertebrates and vertebrates (Gonzalez-Wevar et al. 2018, Maturana et al. 2021, Rozzi et al. 2022). Therefore, in terms of biodiversity, the Subantarctic area in both terrestrial and marine environments still represents a knowledge gap compared even to Antarctica (Rozzi et al. 2012, Aldea et al. 2020, Contador et al. 2020). In summary, it is critical to fill gaps to generate and digitise open and accessible biodiversity databases across the different ecosystems and taxa.

The Chilean Institute of Biodiversity of Antarctic and Subantarctic Ecosystems (BASE) decided to contribute with the digitisation of the historical sampling fieldwork conducted by the authors across Antarctic, Subantarctic and Magellanic Subantarctic ecoregions. This effort will increase the biodiversity data available for these latitudes and improve our understanding of the magnitude of the potential effects of biodiversity loss.

## General description

### Purpose

These data aim to comprehensively describe the geographic distribution of aquatic macroinvertebrates in southern South America, Subantarctica and Antarctica ecoregions. Although this database only included four phyla - and therefore is not completely representative of the extant biodiversity in those regions - we aim to expand the work to include more phyla, species and geographic coverage. The development and continuously updated database will improve our knowledge of Antarctic and Subantarctic biodiversity and initiate long-term biodiversity monitoring across marine and terrestrial ecosystems to detect significant modifications due to global change.

## Project description

### Title

State of Antarctic and Subantarctic biodiversity

### Personnel

Julieta Orlando, Claudia Maturana, Sebastián Rosenfeld, Melisa Gañán, Javier Rendoll

### Study area description

Antarctic and Subantarctic ecoregions, including the Cape Horn Biosphere Reserve (CHBR)

### Funding

ICM-ANID Program ICN2021_002

## Sampling methods

### Sampling description

Since the sampling was carried out by various groups of researchers from different ecosystems and habitats, the sampling methodology presents variations related to the field team and the particular conditions of each collection site. Additionally, each group of taxa requires different sampling techniques; however, here we present the two principal methodologies for collection of aquatic invertebrates.

**Marine**

The specimens were collected following a previous methodology (Rosenfeld et al. 2022). In particular, the different taxa were collected using three methods: 1) manual collection in the intertidal zone, with molluscs being sampled individually; 2) SCUBA diving between 1 and 15 m depth, where the sea urchins were manually collected; and 3) SCUBA diving between 1 and 20 m depth, where the substrates (e.g. sediments, macroalgae) were collected. Rock substrates were subsequently scraped to ensure that all species and specimens were collected. Each macroalga sample was placed in a plastic bag. After collection, specimens were kept alive and transported on-board or to the research station. Each sample was then gently agitated to detach the associated fauna.

For intertidal insect sampling, the intertidal area was surveyed during low tide and adult individuals were collected using an entomological aspirator (insect's pooter).

**Freshwater**

Individuals were collected from lakes, ponds and other freshwater bodies using a Surber net of 0.09 m^2^ area and 243 μm mesh. A Malaise trap was also placed at each site to obtain adult representatives of the organisms needed to identify them to the lowest possible taxonomic level. Traps were placed in the late afternoon and left overnight at each site.

### Quality control

All captures were identified up to species level following available taxonomic keys for the different groups. GPS positions were recorded for each sample location. Specimens were immediately preserved in ethanol (95%) to be transported to the laboratory. The specimens belonging to other phyla not included in this study were kept in ethanol 95%, classified by phylum waiting to be described and digitised.

## Geographic coverage

### Description

**Figure 1.**
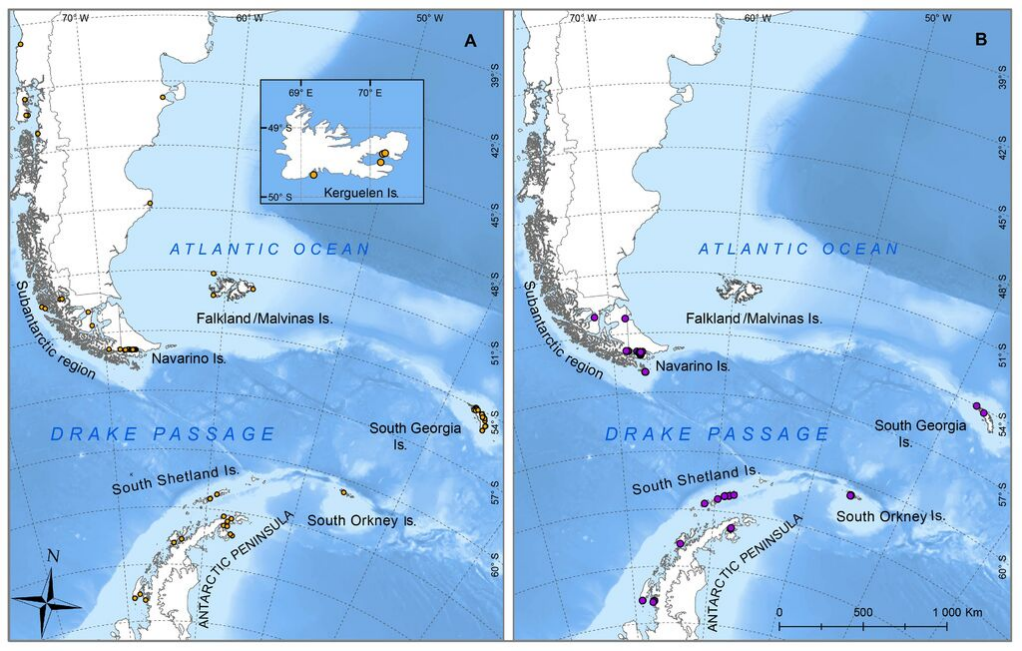
Occurrence of marine (yellow circles) and freshwater (purple circles) invertebrates throughout all sampling sites.

The geographic extent of the digitised dataset is placed in the following biogeographic areas (Fig. 1: 1) the southern part of the Magellan Province, including the Cape Horn Biosphere Reserve (CHBR); 2) the Subantarctic island of South Georgia; 3) the South Orkney Islands; 4) the South Shetland Islands; 5) the West and East Antarctic Peninsula and 6) Kerguelen Island.

### Coordinates

68 and 39 Latitude; 74 and 72 Longitude.

## Taxonomic coverage

### Description

Morphological observations were performed under an OLYMPUS stereomicroscope CX31. Taxonomic identification of the molluscs was based on a complete study of the current literature (e.g. Troncoso et al. (2001), Aldea and Troncoso (2008), Engl (2012), Ibáñez et al. (2019)), as well as on classic works (Smith 1879, v Martens and Pfeffer 1886, Strebel 1908, Powell 1954) and systematics studies about specific taxa (Zelaya 2004, Pastorino 2005, Zelaya 2005, Pastorino 2016, Zelaya et al. 2020). The identification of echinoidea was based on Larrain (1975).

For specific taxonomic identifications in the Arthropoda database, we followed the available taxonomic keys (Ephemeroptera: Domínguez and Fernández (2009); Coleoptera: Libonatti et al. (2011); Plecoptera: Nieto 2004, McLellan and Zwick (2007); Branchiopoda: Rogers et al. (2008); Odonata: von Ellenrieder (2003)). For general freshwater macroinvertebrates identification, we used available taxonomic guides (Merritt and Cummins 1996, Domínguez and Fernández 2009).

**Figure 2.**
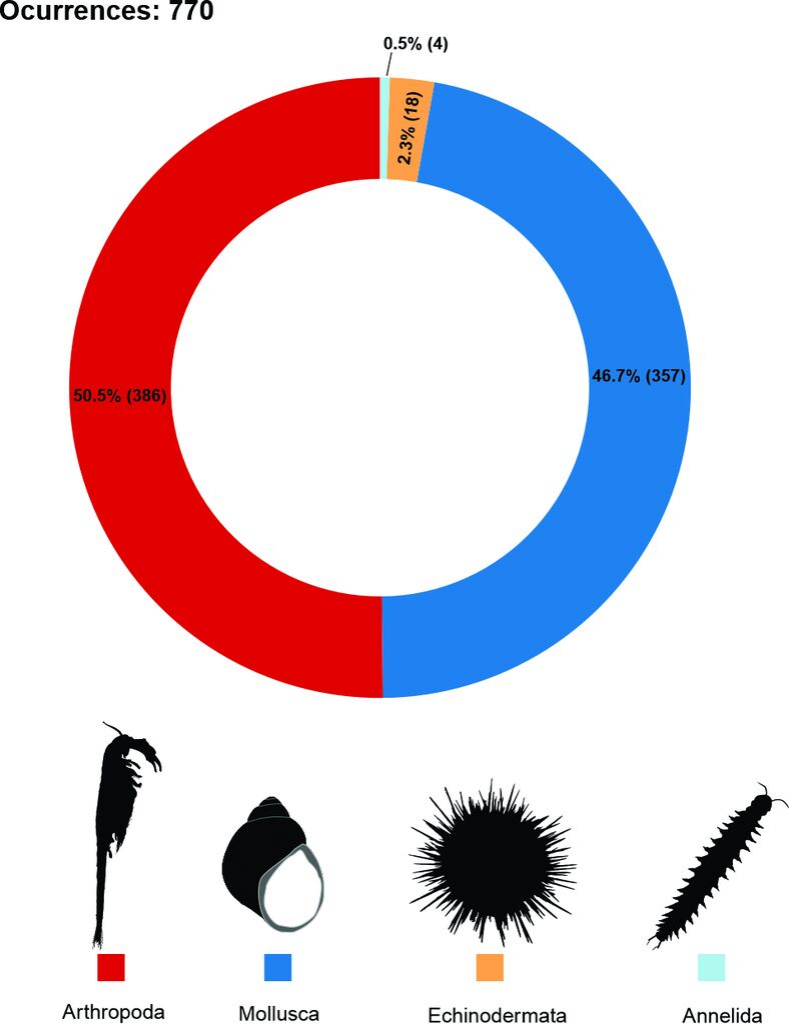
Representation of total occurrences from four phyla. Data were collected from different expeditions to the Antarctic and Subantarctic regions. The absolute values of occurrences are represented in parentheses.

The taxonomic coverage of the dataset consists of all captures of aquatic invertebrates from marine (sub- and intertidal) and freshwater (rivers, lakes and lagoons) habitats. This database comprises 770 occurrences across four invertebrate Phyla: Arthropoda, Annelida, Mollusca and Echinodermata. Of the total occurrences, 50.1% corresponded to Arthropoda, 47% to Mollusca, 2.3% to Echinodermata (1 species) and 0.5% to Annelida (1 taxon at genus level) (Fig. 2). The databases will be described below based on the type of environment (marine or freshwater) and discussed, based on the most representative taxa in terms of occurrences.

**Marine**

**Figure 3.**
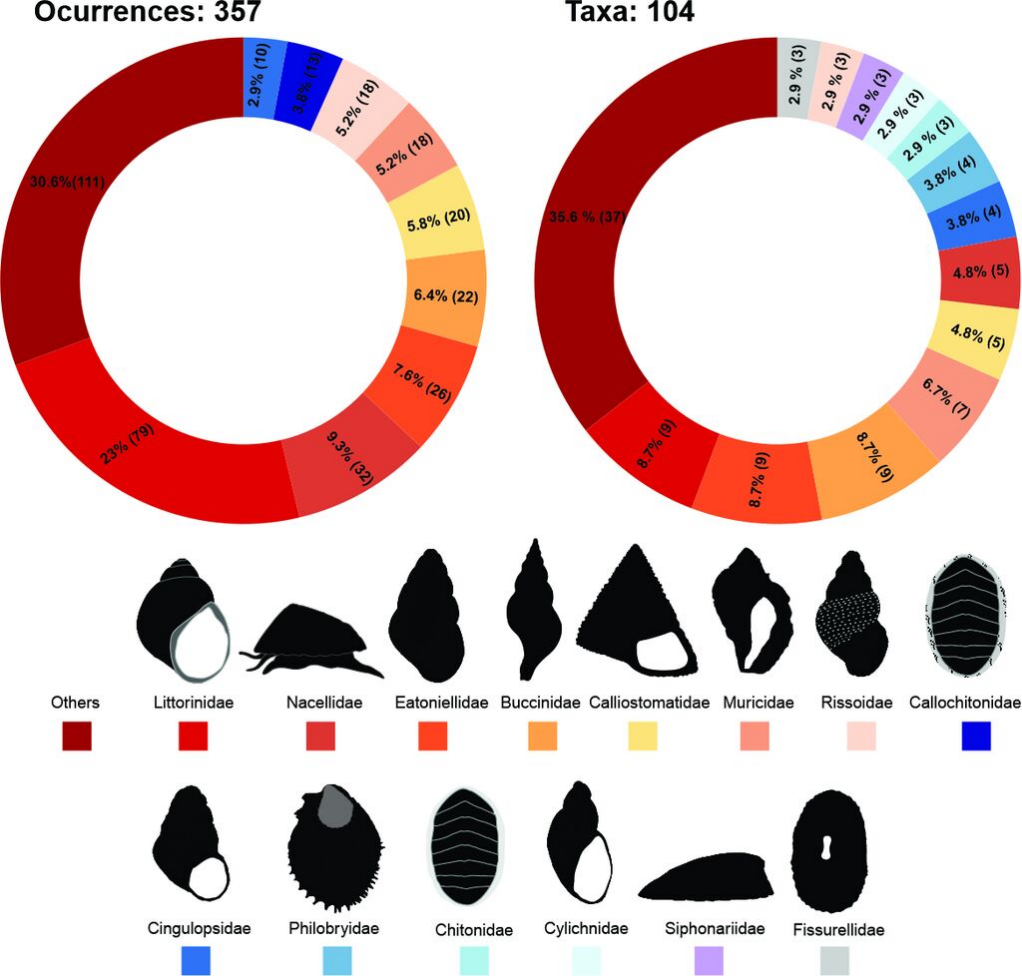
Occurrences and total taxa by families from Mollusca database. Data were collected from different expeditions to the Antarctic and Subantarctic regions. The absolute values of occurrences and species are represented in parentheses.

Considering all the revised specimens, the mollusc database includes 357 records, representing 104 taxa belonging to the classes Polyplacophora, Gastropoda and Bivalvia. Of the total number of taxa, 93 were identified at the species level, six at the genus level and five were left as "cf" (i.e. conferred) because there was no concrete background to identify it with certainty. Of the total occurrences, 277 are gastropods, corresponding to 76 recorded taxa; 40 are chitons, belonging to 10 species and 36 are bivalves, belonging to 17 species. The main families in terms of occurrence and number of species were Littorinidae, Eatoniellidae, Nacellidae, Muricidae and Calliostomatidae, accumulating together ~ 51% of the total occurrences and ~ 42% of the total species (Fig. 3).

**Freshwater**

**Figure 4.**
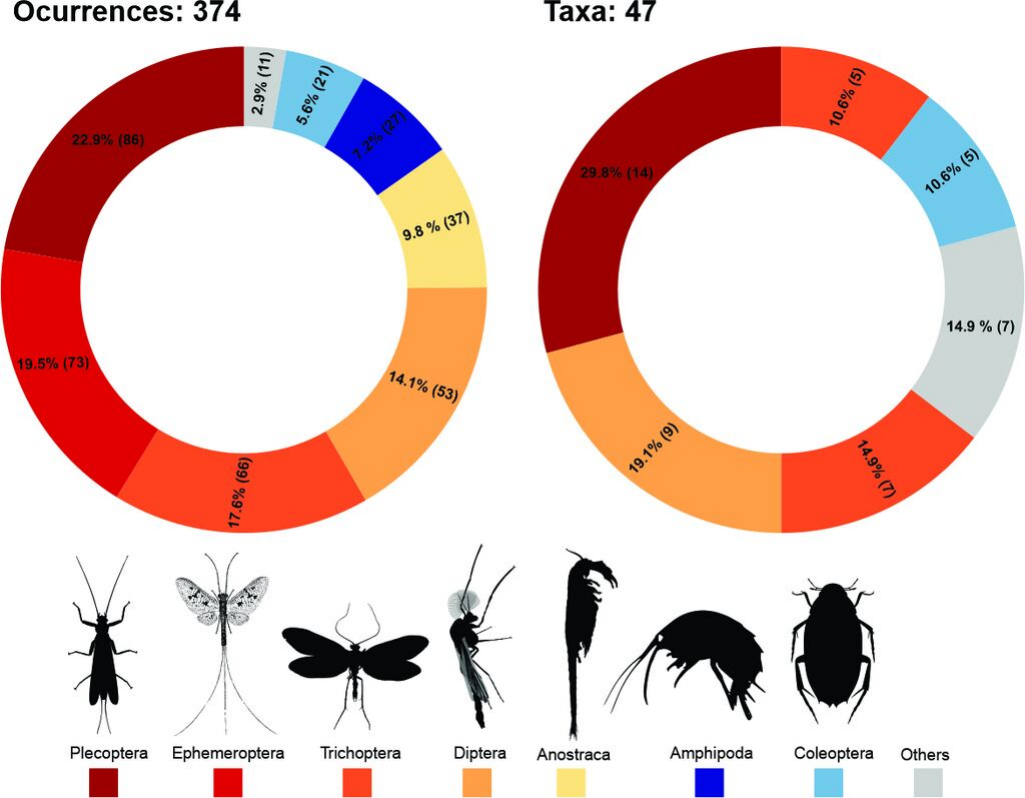
Occurrences and total taxa by orders from the Arthropoda database. Data were collected from different expeditions to the Antarctic and Subantarctic regions. The absolute values of occurrences and species are represented in parentheses. Credits for images Maxime Dahirel (coleoptera) and Didier Descouens vectorised by T. Michael Keesey (trichoptera).

The freshwater ecosystems included three phyla: Annelida, Mollusca and Arthropoda, the latter being the most complete in terms of taxonomic coverage, occurrences and geographic extent. Considering all the Arthropoda database, we included 374 records, from which Insecta was the most representative class (305 records), followed by Branchiopoda (38), Malacostraca (27) and Maxillopoda (4). Within the total records, we detected 48 taxa, from which 25 were identified at the species level and 23 at the genus level. We have detailed the most diverse orders regarding occurrences and taxonomic diversity (Fig. 4).

### Taxa included

**Table:** 

Rank	Scientific Name	
species	*Tonicinazschaui* (Pfeffer, 1886)	
species	*Leptochitonkerguelensis* Haddon, 1886	
species	*Callochitonsteinenii* (Pfeffer, 1886)	
species	*Callochitonpuniceus* (Gould, 1846)	
species	*Toniciachilensis* (Frembly, 1827)	
species	*Tonicialebruni* Rochebrune, 1884	
species	*Chitonmagnificus* Deshayes, 1827	
species	*Plaxiphoraaurata* (Spalowsky, 1795)	
species	*Nuttallochitonmartiali* (Rochebrune, 1889)	
species	*Ischnochitonstramineus* (G. B. Sowerby I, 1832)	
species	*Hemiarthrumsetulosum* Carpenter [in Dall], 1876	
species	*Fissurellideapatagonica* (Strebel, 1907)	
species	*Fissurellaoriens* G. B. Sowerby I, 1834	
species	*Puncturellaconica* (d'Orbigny, 1841)	
species	*Scurriaceciliana* (d'Orbigny, 1841)	
species	*Nacellaconcinna* (Strebel, 1908)	
species	*Nacelladeaurata* (Gmelin, 1791)	
species	*Nacellaflammea* (Gmelin, 1791)	
species	*Nacellamytilina* (Helbling, 1779)	
species	*Nacellamagellanica* (Gmelin, 1791)	
species	*Margarellaviolacea* (P. P. King, 1832)	
species	*Margarellaantarctica* (Lamy, 1906)	
species	*Margarellaachilles* (Strebel, 1908)	
species	*Margarellasteineni* (Strebel, 1905)	
species	*Margarellatropidophoroides* (Strebel, 1908)	
species	*Munditiameridionalis* (Melvill & Standen, 1912)	
species	*Onobagrisea* (Martens, 1885)	
species	*Onobakergueleni* (E. A. Smith, 1875)	
species	*Subonobaturqueti* (Lamy, 1906)	
species	*Rissoellapowelli* Ponder, 1983	
species	*Pickeniasignyensis* Ponder, 1983	
species	*Crepipatelladilatata* (Lamarck, 1822)	
species	*Laevilacunariaantarctica* (Martens, 1885)	
species	*Laevilacunariabennetti* (Preston, 1916)	
species	*Laevilitorinaantarctica* (E. A. Smith, 1902)	
species	*Laevilitorinaclaviformis* Preston, 1916	
species	*Laevilitorinaumbilicata* Pfeffer, 1886	
species	*Laevilitorinacaliginosa* (Gould, 1849)	
species	*Laevilitorinapygmaea* Pfeffer, 1886	
species	*Laevilitorinavenusta* Pfeffer, 1886	
species	*Pellilitorinapellita* (Martens, 1885)	
species	*Pellilitorinasetosa* (E. A. Smith, 1875)	
species	*Eatonielladenticula* Ponder & Worsfold, 1994	
species	*Eatoniellanigra* (d'Orbigny, 1840)	
species	*Eatoniellakerguelenensis* (E. A. Smith, 1875)	
species	*Eatoniellacaliginosa* (E. A. Smith, 1875)	
species	*Eatoniellacontusa* Strebel, 1908	
species	*Eatoniellasubgoniostoma* Strebel, 1908	
species	*Eatoniellastrebeli* Ponder & Worsfold, 1994	
species	*Eatoniellaglacialis* (E.A. Smith, 1907)	
species	*Eatoniellacana* Ponder, 1983	
genus	*Marseniopsis* sp.	
species	Skenella umbilicata Ponder, 1983	
species	*Skenellageorgiana* Pfeffer, 1886	
species	Skenellacf.wareni Ponder & Worsfold, 1994	
species	*Liotellacrassicostata* (Strebel, 1908)	
species	*Sinubersculptum* (E. von Martens 1878)	
species	*Eumetulapulla* (Philippi, 1845)	
species	*Omalogyraantarctica* Egorova, 1991	
species	*Microdisculasubcanaliculata* (E.A. Smith, 1875)	
species	*Acanthinamonodon* (Pallas, 1774)	
species	*Trophonplicatus* (Lightfoot, 1786)	
species	*Trophongeversianus* Pallas, 1774	
species	*Trophonnucelliformis* P. G. Oliver & Picken, 1984	
species	*Trophonbrevispira* E von. Martens, 1885	
species	*Fuegotrophonpallidus* (Broderip, 1833)	
species	*Xymenopsismuriciformis* (P. P. King, 1832)	
genus	*Prosipho* sp.	
species	Prosiphocf.chordatus (Strebel, 1908)	
species	Prosiphocf.gracilis Thiele, 1912	
species	*Prosiphohedleyi* Powell, 1958	
species	*Falsimohniaminor* (Strebel, 1908)	
species	*Chlanidotadensesculpta* (Martens, 1885)	
species	*Pareuthriafuscata* (Bruguière, 1789)	
species	*Microdeuthriamichaelseni* (Strebel, 1905)	
species	*Meteuthriamartensi* (Strebel, 1905)	
species	*Mathildamagellanica* P. Fischer, 1873	
genus	*Flabellina* sp.	
species	*Toledoniapalmeri* Dell, 1990	
species	Toledoniacf.palmeri Dell, 1990	
species	*Toledoniaparelata* Dell, 1990	
species	*Scissurellapetermannensis* Lamy, 1910	
species	*Scissurellaclathrata* Strebel, 1908	
species	*Siphonarialateralis* Gould, 1846	
species	*Siphonariafuegiensis* Güller, Zelaya & Ituarte, 2016	
species	*Siphonarialessonii* Blainville, 1827	
species	*Onchidellamarginata* (Couthouy in Gould, 1852)	
species	*Turbonillastrebeli* Corgan, 1969	
species	*Neobuccinumeatoni* (E. A. Smith, 1875)	
species	*Kidderiasubquadrata* (Pelseneer, 1903)	
species	*Kidderiaminuta* Dall, 1876	
species	*Lissarcamiliaris* (Philippi, 1845)	
genus	*Lissarca* sp.	
species	*Gaimardiatrapesina* (Lamarck, 1819)	
genus	*Philobrya* sp.	
species	*Philobryaquadrata* (Pfeffer in Martens & Pfeffer, 1886)	
genus	*Neolepton* sp.	
species	*Laternulaelliptica* (P. P. King, 1832)	
species	*Limeapygmaea* (Philippi, 1845)	
species	Aequiyoldiacf.eightsii (Jay, 1839)	
species	*Altenaeumcharcoti* (Lamy, 1906)	
genus	*Hiatella* sp.	
genus	*Lasaea* sp.	
species	*Aulacomyaatra* (Molina, 1782)	
species	*Perumytiluspurpuratus* (Lamarck, 1819)	
species	*Arbaciadufresnii* (Blainville, 1825)	
species	*Telmatogetonmagellanicus* (Jacobs, 1900)	
species	*Rhionaeschnavariegata* (Fabricius, 1775)	
genus	*Helobdella* sp.	
species	*Lancetesangusticollis* (Curtis, 1839)	
genus	*Lancetes* sp.	
genus	*Liodessus* sp.	
genus	*Luchoelmis* sp.	
genus	*Haliplus* sp.	
species	*Metamoniusanceps* (Eaton, 1883)	
species	*Massartelopsisirarrazavali* (Demoulin, 1955)	
species	*Meridialarischiloeensis* (Demoulin, 1955)	
species	*Andesiopstorrens* (Lugo-Ortiz & McCafferty, 1999)	
species	*Aubertoperlakuscheli* Illies, 1963	
genus	*Aubertoperla* sp.	
genus	*Antarctoperla* sp.	
genus	*Notoperla* sp.	
species	*Rhithroperlarossi* (Froehlich, 1960)	
species	*Udamocerciaantarctica* (Enderlein, 1905)	
genus	*Udamocercia* sp.	
species	*Austrocosmoecushirsutus* Schmid, 1955	
species	*Monocosmoecushyadesii* (Mabille, 1888)	
genus	*Verger* sp.	
species	*Mastigoptilabrevicornuta* (Schmid, 1958)	
species	*Rheochoremamagellanicum* Flint, 1974	
genus	*Rheochorema* sp	
genus	*Sigara* sp.	
species	*Parochlussteinenii* (Gercke, 1889)	
species	*Gigantodaxrufescens* (Edwards, 1931)	
genus	*Gigantodax* sp.	
genus	*Hexatoma* sp.	
genus	*Hemerodromia* sp.	
species	*Edwardsinadispar* Edwards, 1929	
species	*Gigantodaxigniculus* Coscaron & Wygodzinsky, 1962	
genus	*Limonia* sp.	
genus	*Aphroteniella* sp.	
species	*Andesiopstorrens* (Lugo-Ortiz & McCafferty, 1999)	
genus	*Klapopteryx* sp	
species	*Notoperlafuegiana* (Enderlein, 1905)	
genus	*Pelurgoperla* sp.	
genus	*Teutoperla* sp.	
genus	*Aubertoperla* sp.	
genus	*Metacosmoecus* sp.	
species	*Pisidiummagellanicum* (Dall, 1908)	
species	Pectinidens diaphanum (P.P.King, 1832)	
species	*Hyalellasimplex* (Schellemberg, 1943)	
genus	*Hyalella* sp.	
genus	*Daphnia* sp.	
genus	*Boeckella* sp.	
species	*Andesiopsperuvianus* (Ulmer, 1920)	
species	*Metamoniusanceps* (Eaton, 1883)	
species	*Senzilloidespanguipulli* (Navás, 1928)	
species	*Limnoperlajaffueli* (Navás, 1928)	
species	*Antarctoperlamichaelseni* (Klapálek, 1904)	
species	*Branchinectagaini* Daday, 1910	

## Temporal coverage

### Notes

All available records between 2008 and 2023.

## Collection data

### Collection name

BA

### Specimen preservation method

ethanol 95%

### Curatorial unit

plastic

## Usage licence

### Usage licence

Creative Commons Public Domain Waiver (CC-Zero)

## Data resources

### Data package title

Occurrences of aquatic invertebrates in the Antarctic and Subantarctic regions

### Resource link

https://www.gbif.org/dataset/1eb4dc17-46f3-465e-9846-94e70d15ff78

### Alternative identifiers

http://gbif-chile.mma.gob.cl/ipt/resource?r=aquatic_invertebrates

### Number of data sets

1

### Data set 1.

#### Data set name

Occurrences of aquatic invertebrates in the Antarctic and Subantartic regions

#### Data format

Darwin Core

#### Description

The dataset contains occurrence data from marine and freshwater invertebrates across numerous samplings from 2008 to 2023 (Gañán et al. 2023). From the 770 occurrences, we identified 160 taxa, 125 at species level and 35 at the genus level. A description of column headers used as given below.

**Data set 1.** 

Column label	Column description
occurrenceID	Unique identifier for each occurrence per taxa.
institutionCode	Unique identifier for the institution having custody of the object(s) or information referred to in the record.
collectionCode	The coden identifying the collection from which the record was derived.
catalogNumber	A unique identifier for the record within the dataset.
ocurrenceStatus	The statement about the presence of the Taxon at the given Location.
collectionID	The identifier for the collection or dataset from which the record was derived.
language	The language of the resource.
licence	The legal document giving official permission to share and adapt with appropiate credits.
bibliographicCitation	The bibliographic reference for the resource as a statement indicating how this record should be cited and attributed when used.
taxonRank	The taxonomic rank of the most specific name in the scientificName.
kingdom	The full scientific name of the kingdom in which the taxon is classified.
phylum	The full scientific name of the phylum in which the taxon is classified.
class	The full scientific name of the class in which the taxon is classified.
order	The full scientific name of the order in which the taxon is classified.
family	The full scientific name of the family in which the taxon is classified.
genus	The full scientific name of the genus in which the taxon is classified.
scientificName	The full scientific name in the lowest level taxonomic rank that was determined.
specificEpithet	The name of species epithet of the scientificName.
scientificNameAuthorship	The authorship information for the scientificName formatted according to the conventions of the applicable nomenclaturalCode.
acceptedNameUsageID	An identifier for the documented meaning of the name according to a source of the currently valid (zoological) or accepted (botanical) taxon. We included GBIF and World Register of Marine Species (WoRMS) website codes.
individualCount	The number of individuals present at the time of the Occurrence, if it were countable.
country	The name of the country or major administrative unit in which the Location occurs.
countryCode	The standard code for the country in which the Location occurs following the best practice using an ISO 3166-1-alpha-2 country code.
locality	The specific description of the place in which the collection was made.
island	The name of the island in which the Location occurs.
waterBody	The name of the water body in which the Location occurs. We include best practice to use a controlled vocabulary.
decimalLongitude	The geographic longitude in decimal degrees of the geographic centre of a Location. Positive values are east of the Greenwich Meridian, negative values are west of it. Legal values lie between -180 and 180, inclusive.
decimalLatitude	The geographic latitude in decimal degrees of the geographic centre of a Location. Positive values are north of the Equator, negative values are south of it. Legal values lie between -90 and 90, inclusive.
coordinateUncertaintyInMetres	The horizontal distance in metres from the given decimalLatitude and decimalLongitude describing the smallest circle containing the whole of the Location. We used the reasonable lower limit on or after 01-05-2020 of a GPS.
geodeticDatum	The geodetic datum upon which the geographic coordinates given in decimalLatitude and decimalLongitude are based.
georeferencedBy	A person or a list concatenated and separated of names of people who determined the georeference for the Location.
eventDate	The date-time when the event was recorded. We used best practice using the ISO 8601:1:2019.
year	The four-digit year in which the Occurrence was recorded, according to the Common Era Calendar.
month	The integer month in which the Occurrence was recorded.
minimumDepthInMetres	The lesser depth below the local surface in metres.
maximumDepthInMetres	The greater depth below the local surface in metres.
basisOfRecord	The specific nature of the data record. We used the recommended best practice of one of the Darwin Core classes.
type	The nature of the resource. We used the recommended best practice of one of the Darwin Core classes.
preparations	A list concatenated and separated of preparations and preservation methods for the specimen.
recordedBy	A person or a list of names of people responsible for recording the original Occurrence.
identifiedBy	A person or a list of names of people who assigned the Taxon to the subject.
habitat	A category or description of the habitat in which the Occurrence was recorded.
datasetID	The identifier for the set of data related to the metadata published in GBIF.
datasetName	The name identifying the dataset from which the record was derived. This column is related to the metadata published in GBIF.
occurrenceRemarks	Comments related to the framework of the published records.
associatedReferences	A list concatenated and separated of bibliographic reference of literature associated with the Occurrence.

## Additional information

As with all datasets, there are two main topics that we want to call attention to and discuss.

### Geographic regions and diversity gaps

**Figure 5.**
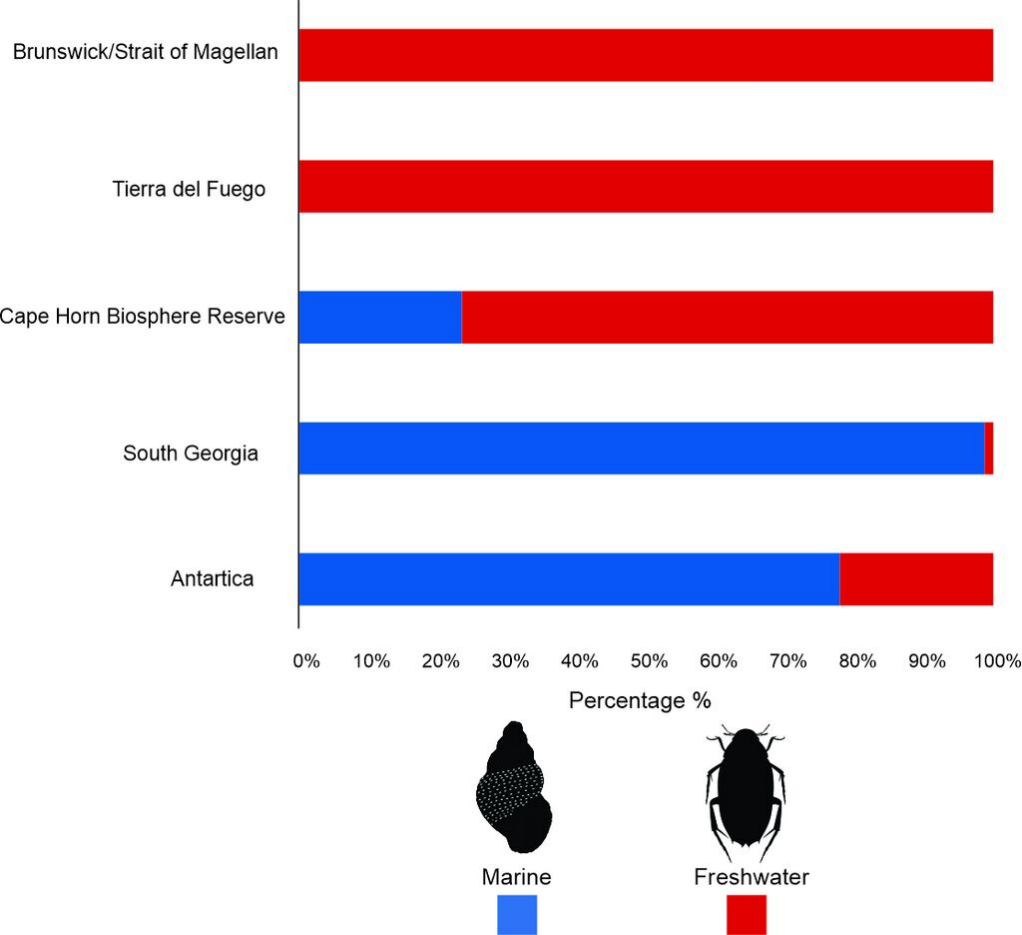
Biodiversity of aquatic invertebrates from the different regions included in this study.

**Figure 6.**
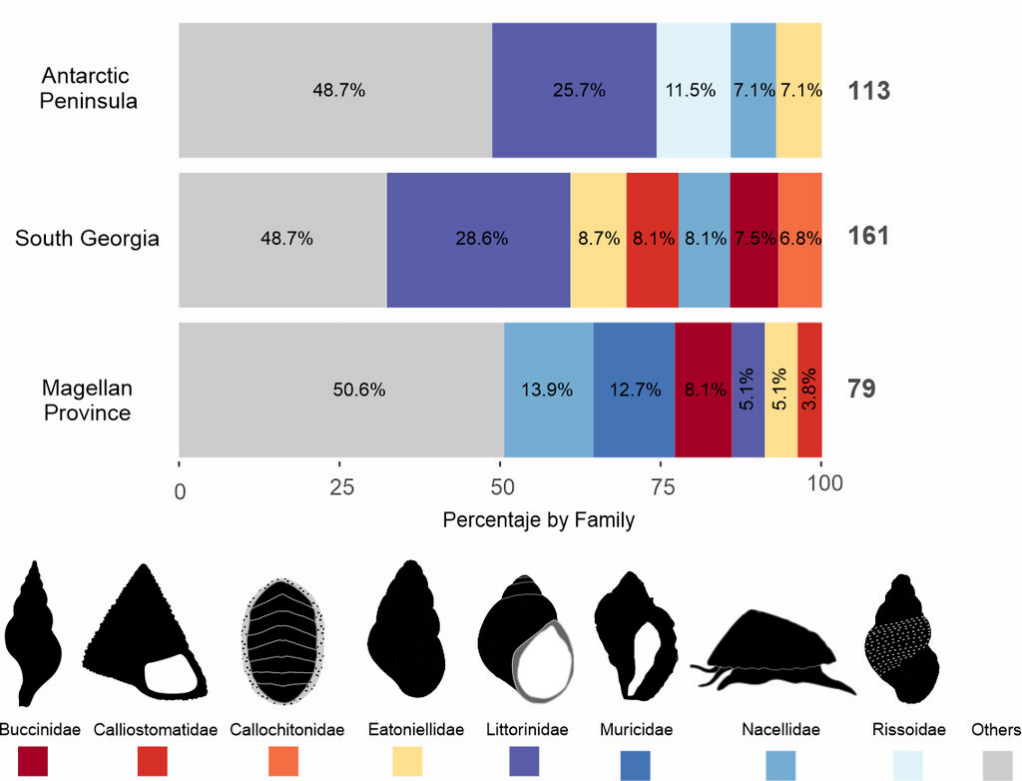
Total occurrences and percentages per dominant molluscs' family. Data were collated from different expeditions to the Antarctic and Subantarctic regions. The absolute values of occurrences per area are represented in bold at the end of each bar.

Within Magellan Province, important differences exist amongst the regions included in this study. For example, CHBR had the highest number of records (440) of freshwater biodiversity overall across all the geographic regions, but in Brunswick/Strait of Magellan and Tierra del Fuego, there is a notorious gap in terms of biodiversity assessment, digitisation of inventories and sampling efforts (Fig. 5). Conversely, South Georgia and Antarctica are very similar in global marine biodiversity, with 166 and 145 occurrences, respectively. However, there are significant differences between marine and freshwater data (Fig. 5) within these regions, the former being the most extensive marine database in these latitudes. In particular, the Littorinidae represented more than 24% in the Antarctic Peninsula and South Georgia; while in the Magellan Province, the families Nacellidae and Muricidae were the most representative, with 13.7% and 12.9%, respectively (Fig. 6).

These results deserve to be highlighted as they point out where sampling and geographic exploration efforts should be aimed and to survey and digitise the under-represented taxa in the territory. In this regard, the highest number of records obtained in the CHBR (Fig. 5) could represent the result of the long-term monitoring of freshwater invertebrates in the area currently conducted by the new Cape Horn International Centre funded by the Chilean Agency of Research and Innovation.

### New records and distribution extensions

**Figure 7.**
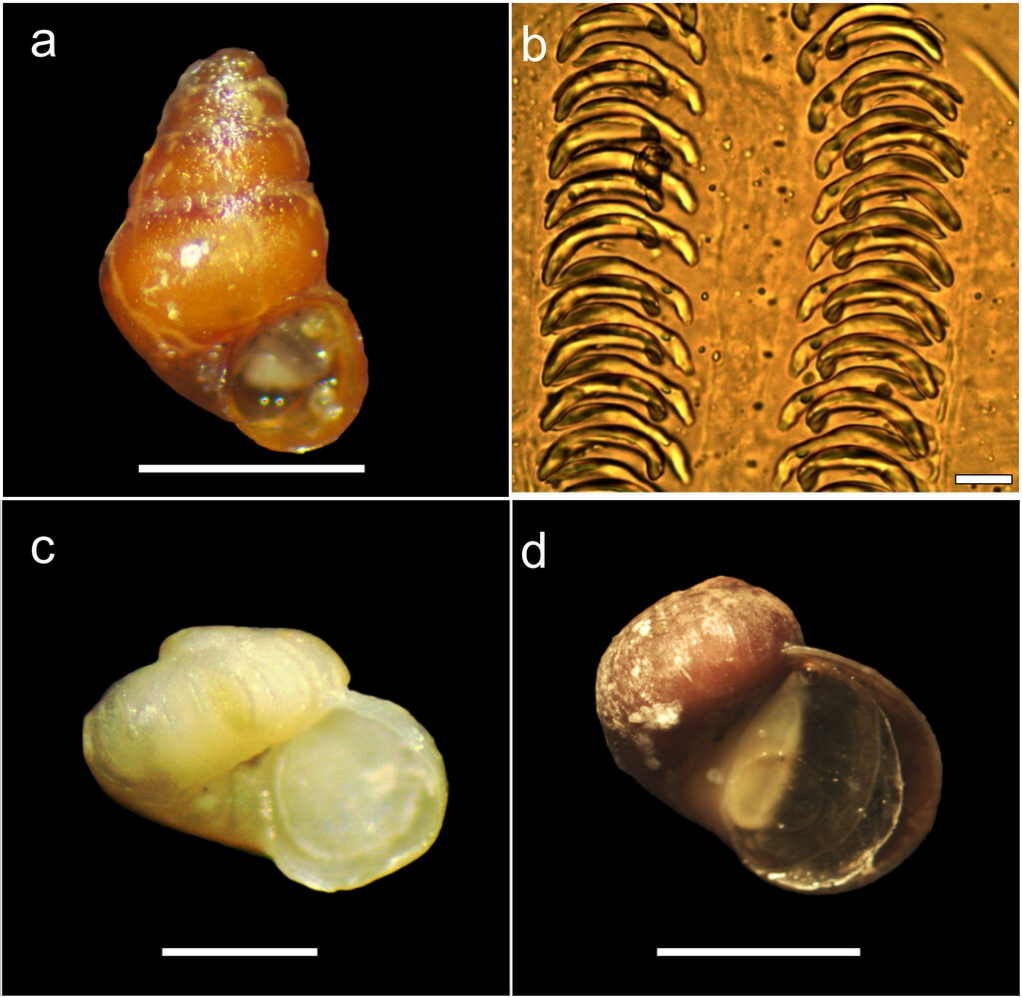
Photographs of new records and distribution extension of marine invertebrates; **a** *Pickeniasignyensis* (bar scale 1 mm); **b** Radula of *P.signyensis* where the absence of the central tooth is shown (bar scale 0.01 mm); **c** *Liotellacrassicostata* (bar scale 0.4 mm); **d** *Laevilacunariaantarctica* Photographs by Sebastián Rosenfeld.

*Pickeniasignyensis* Ponder, 1983 (Fig. 7a) is a micro-gastropod that was described from Borge Bay on Signy Island and, until that time, only recorded for that geographic area (Ponder 1983). This genus is characterszed by the morphological characteristics of its radula, with absence of the central teeth (Ponder 1983, Fig. 7b). This species inhabits areas mainly associated with algae on rocky bottoms and, despite being locally common in Signy, no work on molluscan assemblages associated with macroalgae in the western Antarctic Peninsula has reported this species (Amsler et al. 2015, Martín et al. 2016, Rosenfeld et al. 2017, Amsler et al. 2022). Therefore, this study is the first report of *P.signyensis* outside of South Orkney, specifically in the southern part of the Antarctic Peninsula.

*Liotellacrassicostata* (Strebel, 1908) (Fig. 7c) does not present so many records since its description. It is distributed mainly in the southern part of the Magellan Province, mainly in Tierra del Fuego, Isla de los Estados and Burwood Bank (Strebel 1908, Di Luca and Zelaya 2019). It is characterised by presenting a teleoconch of up to 1.75 whorls, markedly convex in outline, with the last whorl comprising about 90% of the total shell height and a white surface, sculptured with 16 to 19 strong axial ribs per whorl (Di Luca and Zelaya 2019). Our analysed specimens measured approximately 0.7 mm in height and presented 16 axial ribs. This record would represent the first report in the South Georgia Islands.

*Laevilacunariaantarctica* (Martens, 1885) (Fig. 7d) is a species with a restricted distribution in the SO, mainly present in South Georgia Island, South Orkney and the western part of the Antarctic Peninsula (Reid 1989, Engl 2012, Amsler et al. 2015). This work represents the first record of a population of *L.antarctica* in the Weddell Sea, specifically on Cockburn Island, located in front of Seymour Island.

The Antarctic fairy shrimp *Branchinectagaini* Daday, 1910 is an Anostraca species with a distribution from southern South America, Falkland/Malvinas Islands and South Georgia. Within maritime Antarctica, this shrimp occurs from the South Orkney Islands to the southern Antarctic Peninsula, including South Shetland Islands (Hawes 2009). This database contributes new records in the East Antarctic Peninsula (James Ross Island), the southern part of South Georgia and Brunswick Peninsula and Tierra del Fuego from the Magellan Region (see georeferencing information details in GBIF).

Regarding the general freshwater records, all the species found from the Diptera, Rhynchobdellida, Coleoptera, Ephemeroptera, Plecoptera, Trichoptera, Veneroida, Hygrophila, Amphipoda, Cladocera, Calanoida and Basommatophora orders correspond to the southernmost records of their known distribution. In addition, new records of *Parochlussteinenii* (Gercke, 1889) and *Boeckella* were obtained during the last expedition to South Georgia and the Weddell Sea, but these occurrences will be updated into the existing GBIF databases (Maturana et al. 2018, Gañan et al. 2020).

With all this, these new records represent a significant contribution in occurrence data, which undoubtedly contributes to the knowledge of the biodiversity of aquatic invertebrates in the Sub-Antarctic ecoregion of Magallanes, the maritime Antarctic and the Antarctic Peninsula. Additionally, this database allows for contributions to further studies on species distribution, biogeography and ecological niche modelling, amongst others.
